# Virginia Apgar (1909-1974): The Mother of Neonatal Resuscitation

**DOI:** 10.7759/cureus.61115

**Published:** 2024-05-26

**Authors:** Andrew R Ray, Daniel Haines, Ryan Grell

**Affiliations:** 1 Anesthesiology and Perioperative Medicine, University of Louisville School of Medicine, Louisville, USA

**Keywords:** obstetrics, obstetric anesthesia, pediatric resuscitation, apgar score, neonatal resuscitation, anesthesiology

## Abstract

Dr. Virginia Apgar was an American anesthesiologist and researcher who heavily influenced the development of neonatal resuscitation in the immediate postpartum period with her simple five-point scoring system. Today, the APGAR scoring system is used around the world in delivery rooms to guide clinicians in the evaluation of newborns and to distinguish which might need urgent resuscitation. With a simple scoring system, timer, and clipboard, Dr. Virginia Apgar shifted focus from the parturient to the neonate, improving infant mortality as a result.

## Introduction and background

**Figure 1:**
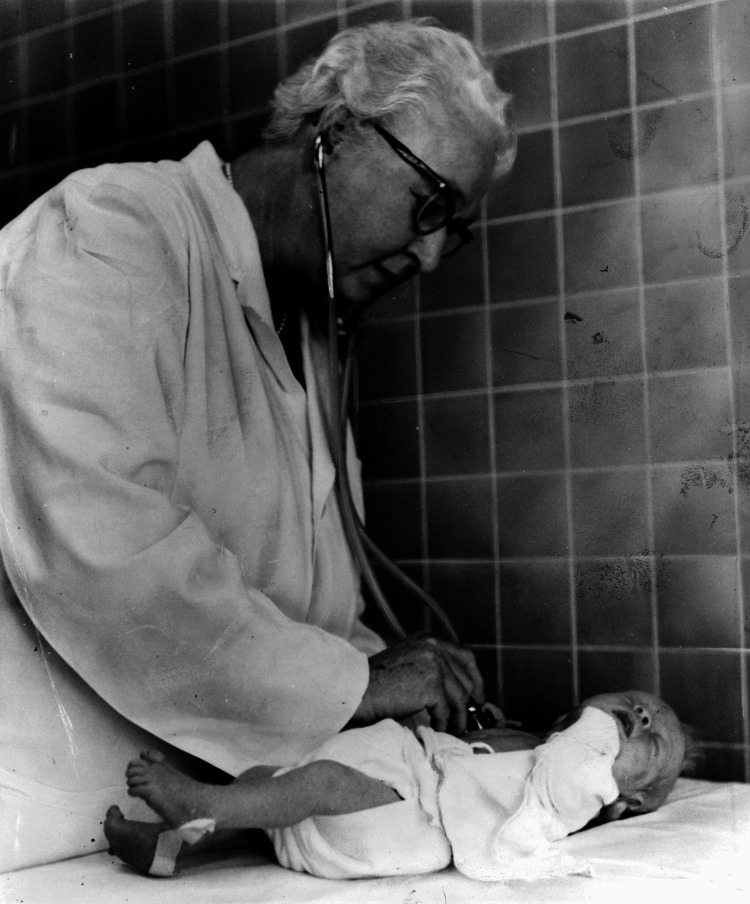
Dr. Apgar examining a neonate Open-source image courtesy of the Library of Congress Prints and Photographs Division, New York World-Telegram, and the Sun Newspaper Photograph Collection. This image is available from the United States Library of Congress's Prints and Photographs division under the digital ID cph.3c31540 [1].

Virginia Apgar is a legendary figure in medicine (Figure 1) [1]. Here, we present Dr. Apgar and her life’s work in developing the APGAR (Appearance, Pulse, Grimace, Activity, and Respiration) score for evaluating newborns and guiding their resuscitation. Apgar’s novel scoring system challenged clinicians to examine neonates for objective signs of distress and sparked the creation of the fields of neonatology and neonatal critical care. Virginia Apgar’s intuitive scoring system started a revolution in obstetrics, pediatrics, and anesthesiology that would save countless lives.

## Review

Early life and career

Virginia Apgar was born in Westﬁeld, New Jersey, US, on June 7, 1909, to parents Charles E. Apgar and Helen May Apgar [2]. From an early age, Virginia Apgar proved she was different than her peers. Demonstrating a near-endless reserve of energy and ambition, Apgar simultaneously participated in several sports teams, served as editor for the school newspaper, and demonstrated a deep passion for Zoology. Apgar also demonstrated a love for the arts by playing the violin and performing in several school theater and drama productions. Apgar went on to further her passion for Zoology at Mount Holyoke College before studying medicine at Columbia University. After medical school, Apgar pursued a surgical residency at New York Presbyterian Hospital where she was mentored by the legendary Allen Whipple. Much to the chagrin of others in the department, Whipple would convince Apgar to pursue a career in anesthesiology because of poor career prospects for female surgeons during the Great Depression. In accepting Whipple’s advice, Apgar went on to complete anesthesiology training at the University of Wisconsin. She would later return to New York Presbyterian and play an instrumental role in establishing its department of anesthesiology. Soon after, she became the first female physician to hold a full professorship at New York Presbyterian's academic affiliate, Columbia University.

In the 1930s, infant mortality was uncomfortably high in America compared with its European peers. Virginia Apgar dedicated her life’s work to solving this problem. Apgar noticed early in her anesthesia training that little clinical attention was focused on the baby in the immediate postpartum period [3-5]. Apgar famously wrote, “Nine months’ observation of the mother surely warrants one-minute observation of the baby [5].” During this period of obstetrics, it was common for junior residents and interns not formally trained in either anesthesiology or resuscitation to be tasked with monitoring the neonate immediately postpartum. Apgar noted that many infants unnecessarily suffered anoxic injuries due to a lack of emphasis on the core tenets of airway management, including clearing obstructive debris from the oropharynx, effective mask ventilation and oxygenation, and intubation [2-5]. These tenets, largely taken for granted in modern anesthesiology and critical care medicine, were revolutionary ideas for the time.

In evaluating the cause of the poor clinical training and education surrounding infant resuscitation, Apgar noticed a general lack of scientific study and agreement on evidence-based practice for resuscitation of the newborn during the immediate postpartum period. This problem was most evident in that there was a general disagreement on what was considered a normal postpartum course for an infant as well as what, if any, warning signs should clinicians be monitoring for in newborns to stratify those who might require additional support. These fundamental gaps in knowledge set the stage for Virginia Apgar’s monumental contribution to anesthesiology, obstetrics, and pediatrics.

The APGAR scoring system for neonates

Dr. Apgar realized that improving infant mortality would require a lot of help. Like many great teachers, she started by educating her residents and students to follow a standardized process when evaluating newborns. According to medical legend, a curious resident once asked Dr. Apgar in the hospital cafeteria what he should be evaluating during his neonatal examination. On a napkin, Apgar scribbled an answer to the resident’s question. In doing so, Apgar created a table of five key indicators of infant health: heart rate, respiratory effort, reflex irritability, muscle tone, and color [2-5]. The presence of the finding would earn the baby two points, while absence received zero points. Any finding noted between these two was assigned one point; therefore, a perfectly healthy newborn would have an Apgar score of 10. Thus, the APGAR score was born (Table 1).

**Table 1:** APGAR scoring system BPM = beats-per-minute

	0 Points	1 Point	2 Points
Appearance (Color)	Cyanotic	Peripheral cyanosis only	Pink/Well perfused
Pulse (Heart rate)	Absent	Less than 100 BPM	More than 100 BPM
Grimace (Reflex irritability)	No response	Grimace or weak cry	Strong cough or cry
Activity (Muscle tone)	Limp	Some flexion	Active motion
Respirations	Absent	Slow and irregular	Strong cry, cough, or sneeze

Dr. Apgar had clear ideas regarding who should administer the examination and when it should be scored. Dr. Apgar strongly discouraged involving the delivering clinician as “he or she is invariably emotionally involved with the outcome of the delivery and with the family, and cannot or unconsciously does not make an accurate decision as to the total score [5]." She continued, "I know of no reliable study which compared scores given by various delivery room personnel, but my impression is strong that obstetricians give higher scores than anesthesiologists, nurse anesthetists, pediatricians or delivery room nurses [5].” Apgar stressed the exam was to take place exactly 60 seconds after delivery. This interval was chosen in 1952 after she evaluated several hundred neonates for the time of maximum clinical depression. Above all, Apgar intended the system to be easy to use and uniform for later scientific study.

Over the next few years, Apgar put her new tool to the test by having her residents apply the scale to 2096 delivery cases at the Presbyterian Hospital in New York [3]. A pattern soon emerged. Dr. Apgar writes “in order to check the approximate accuracy of the various scores, the fate of the infants in poor, fair, and good condition was examined. After this initial experience, it seems to us that groups 8, 9, and 10 indicate infants in good condition; 0, 1, and 2, poor condition; and the remaining scores, fair condition [3].” For the first time, Apgar had demonstrated an objective metric for predicting infant mortality. Apgar’s score also quickly identified babies in distress, thus reducing the time to aggressive resuscitation.

As Dr. Apgar’s data grew to over 15,000 deliveries, so too did support for her system. Drs. Drage and Berendes confirmed the accuracy of Apgar’s score for predicting infant mortality and also improved its precision by expanding the exam to include an evaluation five minutes post-delivery as well [6-8]. Since that addition, one-minute and five-minute APGAR scores have become the cornerstone of newborn evaluations. Apgar and colleagues would also demonstrate a weak correlation of the score with the acid-base status of the infant, which would be criticized and later disproven [9]. The APGAR score also garnered negative attention when misused as a tool to predict neurologic outcomes in newborns [10-12]; however, these applications of Apgar’s scoring system were never its intended use. Apgar’s score was finally vindicated as a key prediction tool for predicting infant mortality and guiding resuscitation when a massive study of 145,627 births again reproduced Apgar’s findings [13].

Late career

In performing thousands of neonatal examinations, Dr. Apgar was exposed to a significant number of neonates with congenital malformations. To improve her statistical knowledge and to be able to more fully explore the correlation between maternal disease and these conditions, Dr. Apgar chose to take a sabbatical from clinical medicine in 1958 to complete a Master of Public Health from the Johns Hopkins School of Public Health [14]. Before even completing her studies, she was approached by the National Foundation for Infantile Paralysis (now the March of Dimes) and offered the opportunity to apply her new knowledge in the position of chief of its new Division of Congenital Malformations. As part of her role, she regularly appeared on talk shows and news stations and authored numerous books spreading sound scientific knowledge to expectant mothers. She was also an early advocate for the utilization of Rh immune globulin (RhoGAM) in multi-parturients to prevent hemolytic anemia in newborns due to Rh incompatibility.

Her final achievement was in helping to create the first Committee on Perinatal Health incorporating every stakeholder in neonatal health, including the American Medical Association, the American College of Obstetricians and Gynecologists, the American Academy of Pediatrics, and the March of Dimes, to improve maternal-fetal health and to further reduce infant mortality [15]. Unfortunately, Dr. Apgar did not survive to see the final report from the committee as she succumbed to liver failure in 1974 in Columbia's New York Presbyterian Hospital, the very hospital where she began her prolific career.

## Conclusions

Dr. Virginia Apgar will be remembered for devising a simple scoring system for evaluating newborns. Apgar’s score is still used globally to help clinicians support babies at risk and predict infant mortality. In fact, it's so universally utilized that it is said that every baby born in a hospital anywhere in the world is first seen through the eyes of Dr. Virginia Apgar. Dr. Apgar forever changed anesthesiology, obstetrics, and pediatrics by achieving her goal of shifting focus to the newborn, saving countless lives along the way.
